# Breathing, postural stability, and psychological health: a study to explore triangular links

**DOI:** 10.3389/fbioe.2024.1347939

**Published:** 2024-04-02

**Authors:** Simone Tassani, Paula Chaves, Marc Beardsley, Milica Vujovic, Juan Ramírez, Jimena Mendoza, Marta Portero-Tresserra, Miguel Angel González-Ballester, Davinia Hernández-Leo

**Affiliations:** ^1^ Department of Information and Communication Technologies Universitat Pompeu Fabra, Barcelona, Spain; ^2^ Technische Universität Wien, Vienna, Austria; ^3^ Universidad Nacional de Colombia, Medellín, Colombia; ^4^ Universidad Iberoamericana, Mexico City, Mexico; ^5^ Universitat Autònoma de Barcelona, Barcelona, Spain; ^6^ Catalan Institution for Research and Advanced Studies (ICREA), Barcelona, Spain

**Keywords:** breathing, posture, stability, psychological health, emotion, Wellbeing

## Abstract

**Objective:**

This study aims to test the hypothesis that breathing can be directly linked to postural stability and psychological health. A protocol enabling the simultaneous analysis of breathing, posture, and emotional levels in university students is presented. This aims to verify the possibility of defining a triangular link and to test the adequacy of various measurement techniques.

**Participants and Procedure:**

Twenty-three subjects (9 females and 14 males), aged between 18 and 23 years, were recruited. The experiment consisted of four conditions, each lasting 3 minutes: Standard quiet standing with open eyes 1), with closed eyes 2), and relaxed quiet standing while attempting deep abdominal breathing with open eyes 3) and with closed eyes 4). These latter two acquisitions were performed after subjects were instructed to maintain a relaxed state.

**Main Outcome Measures:**

All subjects underwent postural and stability analysis in a motion capture laboratory. The presented protocol enabled the extraction of 4 sets of variables: Stabilometric data, based on the displacement of the center of pressure and acceleration, derived respectively from force plate and wearable sensors. Postural variables: angles of each joint of the body were measured using a stereophotogrammetric system, implementing the Helen Hayes protocol. Breathing compartment: optoelectronic plethysmography allowed the measurement of the percentage of use of each chest compartment. Emotional state was evaluated using both psychometric data and physiological signals. A multivariate analysis was proposed.

**Results:**

A holistic protocol was presented and tested. Emotional levels were found to be related to posture and the varied use of breathing compartments. Abdominal breathing proved to be a challenging task for most subjects, especially females, who were unable to control their breathing patterns. In males, the abdominal breathing pattern was associated with increased stability and reduced anxiety.

**Conclusion:**

In conclusion, difficulties in performing deep abdominal breathing were associated with elevated anxiety scores and decreased stability. This depicts a circular self-sustaining relationship that may reduce the quality of life, undermine learning, and contribute to muscular co-contraction and the development of musculoskeletal disorders. The presented protocol can be utilized to quantitatively and holistically assess the healthy and/or pathological condition of subjects.

## 1 Introduction

Modern society is suffering from contemporary pathologies that healthcare institutions can only partially address. Conditions and diseases traditionally linked to aging, including back pain, neck pain, and even arthrosis, are now emerging earlier in life, even during youth and adolescence (Prins et al., 2008). The rising incidence of musculoskeletal pain among younger populations is alarming, not only due to its early onset but also because of the potential to evolve into chronic musculoskeletal pain syndromes that persist into adulthood (Brattberg, 2004; Prins et al., 2008). Emerging evidence from the literature underscores the causal role of emotional distress, already related to the development of mental disorders, in the development of musculoskeletal disorders (Brattberg, 2004; Diepenmaat et al., 2006; McFarlane, 2007). Throughout our lives, we inevitably encounter various stressful situations, some of which can be avoided or mitigated, while others are an inherent part of life. Nevertheless, learning to regulate our emotional response becomes crucial for our adaptation in the environment, health, and wellbeing. Particularly, young individuals, including students, often confront stressors without the appropriate self-regulation skills (Prins et al., 2008; Sawatzky et al., 2012). Moreover, they are prone to adopting sub-optimal musculoskeletal strategies, such as maintaining poor sitting postures (Yang et al., 2020). These psychological and physical aspects, in turn, can impact their learning process and performance (Swiecki et al., 2019). Therefore, there is a need for methods that empower students to enhance their stress management skills, mitigating the potential long-term effects on both their musculoskeletal health and learning outcomes.

However, the direct correction of postural or psychological problems presents challenges. From a physical standpoint, learning correct posture is problematic due to the numerous degrees of freedom that need to be controlled, making it difficult to self-learn good posture (Sheikhhoseini et al., 2018). Technology-based solutions aimed at identifying and helping to correct poor postures have been developed (Wong and Wong, 2008; Byeon et al., 2020). However, the widespread availability of these approaches to all students remains an important challenge. On the psychological side, problems can arise even in earlier phases, with students often avoiding asking for help (Abdollahi et al., 2017). Moreover, students with mental health problems report lower engagement in campus activities (Byrd and McKinney, 2012), and few students experiencing stress-related mental health problems receive treatment (Garlow et al., 2008).

Breathing techniques offer an accessible approach to address both postural and psychological challenges (Gilbert, 2003; Busch et al., 2012) acting as a mediator between the two spheres. Unlike most physiological functions, breathing can be modulated voluntarily and serves as an entry point for both physiological and psychological regulation. Accordingly, clinical trials implementing disciplines specifically focused on breathing, like Tai Chi, Qi Gong, and Yoga, are exponentially increasing (Tassani et al., 2019). Repetitive motor tasks and long-lasting training can cause beneficial or disadvantageous postural adaptation with long-term effects on the Postural Control System (De Blasiis et al., 2022). The main advantages of these breathing practices are that they can be easily taught, are non-pharmacologic, self-administered, come at no cost, and can be performed at anytime and anywhere.

Scientific literature also suggests a clear relation between breathing and posture. Breathing and postural control are mechanically and neuromuscularly interdependent as the main muscles used during respiration (diaphragm and intercostal muscles) also contribute to postural control (Hodges and Gandevia, 2000). Poor posture (as an inclined position) impedes the proper function of the diaphragm resulting in increased activity of the upper respiratory duct (Kim et al., 2017). The effect of breathing on psychological stress is also increasingly studied (Perciavalle et al., 2017; Hopper et al., 2019) and specific breathing patterns have been found to lower physiological arousal associated with emotional distress and measures of state anxiety (Balban et al., 2023).

These findings suggest a tight link between psychological health, breathing, and posture through self-regulation. However, the actual triangular relation between posture, breathing, and stress is still unclear. Understanding this relationship could shed more light on the efficiency of breathing therapies for both aiding young adults in controlling their mental distress and predicting/preventing the development of several musculoskeletal disorders for the population of any age. In this regard, instrumentation methods for the quantitative evaluation of posture, breathing, and stress could be useful to objectively demonstrate this triangular link. In particular, the gold standard for three-dimensional analysis of posture is 3D stereophotogrammetry, an optoelectronic system that allows for the evaluation of the whole-body in standing (Chiari et al., 2002; Rocchi et al., 2004) and while walking (Cappozzo et al., 2005) by using infrared cameras and reflective skin markers placed on specific landmarks. Moreover, 3D stereophotogrammetry is also used for optoelectronic plethysmography to assess thoracic and abdominal movements during breathing (Massaroni et al., 2017). For the analysis of body oscillations, force plate, and stabilometry are used to analyze postural stability parameters in open (De Blasiis et al., 2023) and closed eyes (Fullin et al., 2022), considering their reliability and variability too. Eventually, levels of psychological stress can be evaluated by wearable sensors. Examples include an “in-ear sensor” (Ellebrecht et al., 2022) which measures body temperature and heart rate as well as stabilometric parameters, and a “galvanic skin response (GRS) device” (Ellebrecht et al., 2022), which measures the conductivity of human skin through the activity of sweat glands stimulated by the sympathetic nervous system (SNS).

Currently, there seems to be an absence of studies considering the three effects at the same time. Therefore, the present study aims to test the relationship between breathing, postural stability, and psychological health under the hypothesis that deep abdominal breathing can reduce stress and increase stability in university students. For this reason, a protocol allowing simultaneous analysis of breathing, posture, and emotional levels is presented, to verify the possibility of defining a triangular link and to test the adequacy of different measurement techniques.

## 2 Materials and methods

### 2.1 Recruitment

Twenty-three subjects were recruited for this study, 9 females and 14 males, with ages ranging between 18 and 23 years old, 174 ± 9 cm height, 67.2 ± 9.5 kg weight, and 22.1 ± 2.3 kg/m^2^ of BMI. Twenty-one subjects were within the normal BMI range (18.5–24.9) while two subjects were slightly overweight. No obese or underweight subjects were involved in the study. The inclusion criteria for the study were that the subjects were university students in the first to the third year of their undergraduate studies. Excluded from the study were frequent smokers (those who smoke more than 3 times a week), those with prior musculoskeletal disorders, those with reported cases of anxiety, and those who reported expert knowledge of breathing techniques. All subjects gave informed written consent before participating in the study.

### 2.2 Data collection

For all subjects, three sets of data collection were performed: breathing, postural/stability, and emotional.

#### 2.2.1 Breathing and posture

Breathing and body postural/stability recordings were performed at the Motion Capture Laboratory using optoelectronic technology. Eight infrared cameras were used (BTS Smart-DX 700, 1.5Mpixels 250 fps). Four were placed on tripods close to the subject, with two in front of the subject and the other two behind. The remaining four cameras were fixed to the corners of the ceiling of the room. Two video cameras (BTS VIXTA 50HZ) were placed to record the frontal and the left side of subjects during all data acquisition as a reference for reflective marker positioning over subjects. Further, a force plate (BTS P-6000 50 Hz sampling) was used to acquire stability data. Synchronized data acquisition was guaranteed using the software SMART Capture, developed by BTS Bioengineering.

During posture and breathing analysis, a total of 105 reflective markers were attached to the subjects’ skin of as two different marker protocols were combined. Firstly, breathing data was acquired using a validated protocol implemented by BTS in the software Smart Analyzer that was used for the data analysis (Aliverti and Pedotti, 2003; De Faria Júnior et al., 2013). The protocol consists of eighty-nine reflective markers placed over the chest of subjects and requires subjects to be bare chest. A minor modification to the protocol allowed women to wear any kind of upper undergarment during this study. Secondly, posture was measured using the Helen Hayes protocol with medial markers (Davis et al., 1991). This protocol makes use of 22 reflective markers. However, the placement of some markers coincides in the two protocols. Specifically, markers placed in the shoulders, C7 vertebrae, sacrum, and left and right anterior superior iliac spine were reused. Therefore, only sixteen additional markers were placed on the legs.

**FIGURE 1:**
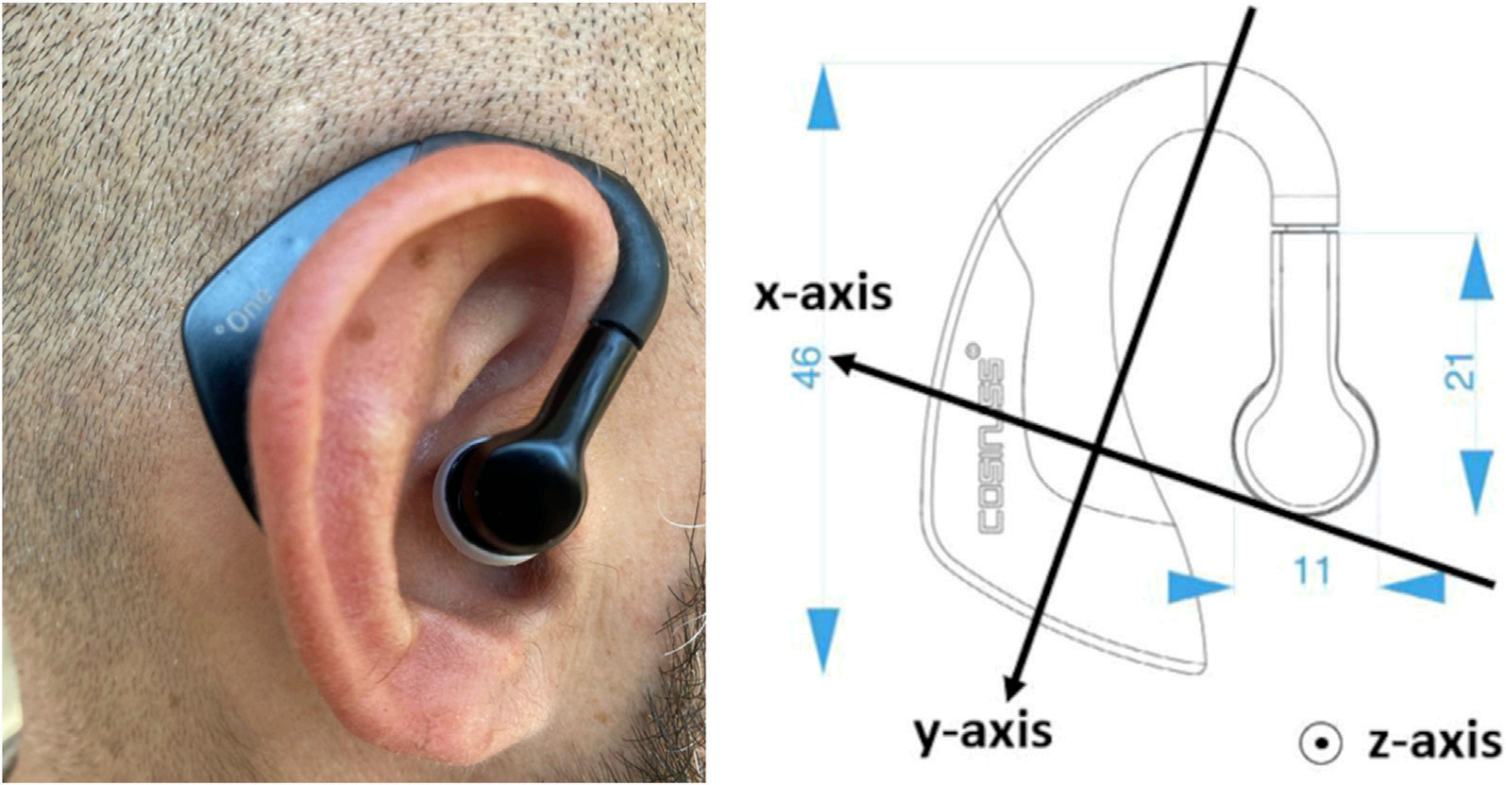
In-ear sensor “Cosinuss One.” On the right are displayed the acceleration directions of the sensor.

Stability estimations were performed by measuring the displacement of the center of pressure (COP) over the force plate. Subjects wore a single 3D commercial accelerometer in-ear sensor, called Cosinuss◦ One, Cosinuss GmbH, Munich, Germany (Burgos et al., 2020). The three axes of the in-ear sensor are identified in Figure 1. The three-axial accelerometric data were also used as estimators of subject stability.

#### 2.2.2 Emotional data

Emotional data were collected following two different approaches. Firstly, psychometric data were used to acquire baseline information and collect subjects’ feelings. Secondly, physiological signals were collected throughout to measure biometrics related to subjects’ emotional responses.

##### 2.2.2.1 Psychometric data

During recruitment, applicants to the study were asked to complete an online survey administered in the format of a Google form. The survey included the State-Trait Anxiety Inventory (STAI—link), Rosenberg Self-Esteem Scale (RSE), and Five Facet Mindfulness Questionnaire. Subjects were also asked some general questions about their knowledge of breathing techniques and questions related to the exclusion criteria.

##### 2.2.2.2 Physiological signals

The same in-ear device used for measuring postural stability (Cosinuss◦ One, Cosinuss GmbH, Munich, Germany) also allowed for the measurement of body temperature and heart rate which were used as estimators of anxiety. Finally, galvanic skin response (GSR) was measured with a device (Shimmer3, Shimmer Sensing, Dublin, Ireland) placed on the arm with connected electrodes positioned close to the palm on the internal side of the wrist.

### 2.3 Procedure

The experiment consisted of four conditions with each recording having a duration of 3 minutes. 1) Standard quiet standing with open eyes and 2) standard quiet standing with closed eyes were the two first recordings. For these conditions, subjects were asked to stand on the force plate in a normal stance that they felt comfortable with and to avoid sudden movements. After the standard condition, subjects were given instructions on performing deep abdominal breathing. They were instructed to breathe in slowly and deeply into the abdomen and to breathe out in a relaxed manner. Subjects performed from six to ten deep breaths with the lab technician while using proprioceptive input in which subjects placed one hand on their chest and the other on their abdomen. Subjects were asked to practice this breathing technique for a few minutes and to perform some exercises to relax their joints (circular motions, flex-extension, ab-adduction, and rotation of each joint: neck, shoulders, hips, knees, and ankles) while maintaining their deep breathing as described in the literature (Tassani et al., 2019). At the end of the relaxation phase, 3) a recording with open eyes and then 4) with closed eyes was taken in which subjects were asked to be in a relaxed state while trying to perform deep breathing. This is what was referred to as the relaxed state.

### 2.4 Data processing

#### 2.4.1 Stability data

Displacement of the center of pressure (COP) was analyzed using the software Sway (BTS Engineering, Milan, Italy). Analysis time was normalized to 180 s and all parameters described in the table below were computed (Table 1).

**TABLE 1:** Complete list of variables analyzed for the stability analysis. Underlined items denote variables not used in the final analysis, after feature reduction.

Stability parameters
Time-domain: Center of Pressure (COP) stabilogram
Transversal COP displacement [mm] (mean and sd)
Longitudinal COP displacement [mm] (mean and sd)
Radius [mm] (mean and sd): COP distance from the barycenter
Maximum and minimum radius [mm]
Transversal and Longitudinal range [mm]
LFS1 [cm]: Length as a function of surface
Equivalent area [mm^2^]: Area marked out by the COP
Equivalent radius [mm]: Radius of the circle with an area equal to the Equivalent Area
Speed [mm] (mean and sd): COP displacement velocity
Inertial axes [mm] X: X-axis of the 95% confidence ellipse area
Inertial axes [mm] Y: *Y*-axis of the 95% confidence ellipse area
Regression angle [°]: Inclination of the *X*-axis of inertia with respect to the *x*-axis of the reference system
Variable mean area [mm^2^]
Variable sum area [mm^2^]
Time-domain: Sway density curve
Peak number: number of peaks of the sway density curve
Peak amplitudes [s] (mean and sd): amplitude of the peaks of the sway density curve
Peak times [s] (mean and sd): time between peaks of the sway density curve
Peak distance [mm] (mean and sd): distance between peaks of the sway density curve
Frequency-domain
Px spectrum maximum peak: Max peak of the COP frequency spectrum in the *x* direction
Px spectrum MaxPeak frequency [Hz]: Frequency of the max peak of the spectrum in the x direction
Py spectrum maximum peak: Max peak of the COP frequency spectrum in the *y* direction
Py spectrum MaxPeak frequency [Hz]: Frequency of the max peak of the spectrum in the *y* direction
D spectrum maximum peak: Max peak of the spectrum of the distance from the barycenter
D spectrum Max Peak frequency Hz: Frequency of the max peak of the spectrum of the distance from the barycentre
Px spectrum mean values: Mean amplitude of the spectrum in the x direction
Px spectrum mean values Freq: Mean frequency of the spectrum in the *x* direction
Py spectrum mean values: Mean amplitude of the spectrum in the y direction
Py spectrum mean values Freq: Mean frequency of the spectrum in the *y* direction
D spectrum mean values: Mean amplitude of the spectrum of the distance from the barycenter
D spectrum mean values Freq: Mean frequency of the spectrum of the distance from the barycenter

#### 2.4.2 Body posture

**FIGURE 2:**
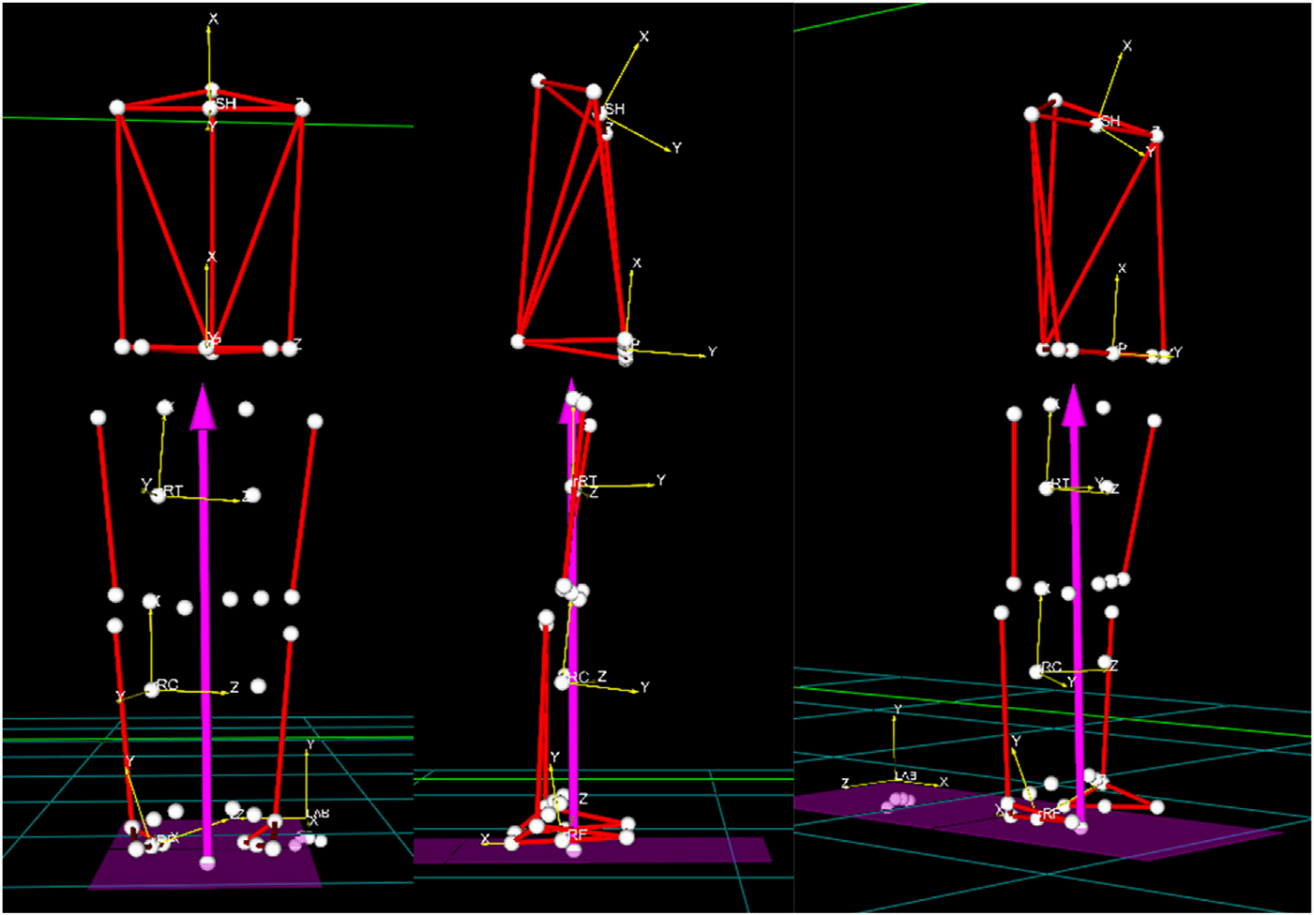
References systems of each body segment.

Body posture analysis was performed using references identified by the Helen Hayes protocol as shown in Figure 2. As a general description, in the reference system of each body segment, X is the longitudinal axis of the segment and points up, Y is the sagittal axis of the segment and points forward, and Z is the transversal axis of the body and points to the left of the subject. The only exception to this definition is the reference system of the foot in which X points backward and therefore Y points up.

These definitions are general for all joints in the body. All measures are relative of the distal segment to the proximal one. In the case of the pelvis and trunk, Rotation refers to when there is rotation around the *X*-axis. Obliquity refers to when the rotation is around the *Y*-axis. Tilt refers to when the rotation is around the *Z*-axis. However, for these two segments, measures are defined with respect to the posture reference system (*X*-axis—vertical direction in the laboratory, *Z*-axis—axis passing through the two markers in the heels, *Y*-axis—vectorial product of X and Z). The complete list of body posture angles and their acronyms is presented in Table 2.

**TABLE 2:** List of postural angles obtained and their acronyms. Underline items denote variables not used in the final analysis, after feature reduction.

Body posture parameters and their acronyms			
Postural angles		Acronyms	
		Right	Left
Spine	Lateral Bending	SPML	
	Rotation	SPIE	
	Flex-Extension	SPFE	
Trunk	Rotation	SHROT	
	Obliquity	SHOBLI	
	Tilt	SHTILT	
Pelvis	Tilt	PTILT	
	Rotation	PROT	
	Obliquity	POBLI	
Hip	Rotation	RHPIE	LHPIE
	Flex-Extension	RHPFE	LHPFE
	Ab-Adduction	RHPAA	LHPAA
Knee	Rotation	RKIE	LKIE
	Flex-Extension	RKFE	LKFE
	Valgus-Varo	RKAA	LKAA
Ankle	Rotation	RFIE	LFIE
	Dorsi-Plantar Flex	RAFE	LAFE
	Prone-supine	RFAA	LFAA
Foot	Foot Progression	RAIE	LAIE

#### 2.4.3 Breathing

**FIGURE 3:**
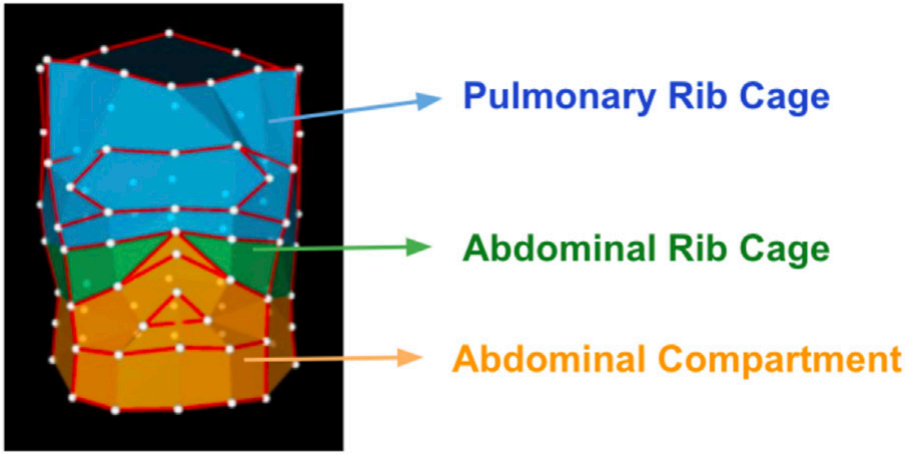
Compartmental 89-marker model of the chest wall in Optoelectronic Plethysmography.

The volumetric geometrical model of the chest wall was run through an OptoElectronic Plethysmography (OEP) protocol for quiet breathing, implemented by BTS Engineering. The protocol computes the chest’s tidal volume and the three thoracic compartments (Pulmonary Rib Cage, Abdominal Rib Cage, and Abdominal) (Figure 3).

### 2.5 Data analysis

Data analysis was performed in consecutive steps that were repeated for each typology of data acquired to test possible relations between abdominal breathing, posture, and emotions. Analyses were performed using SPSS (version 23.0; IBM Corp., Armonk, NY, United States).

Firstly, stability data were normalized by the height of subjects as suggested in the literature (Chiari et al., 2002). Normalization was not applied to postural parameters, which are angles and not affected by subject height, nor to emotional data. Breathing data were not normalized either since the analysis focussed on the percentages of use of each thoracic compartment.

The use of each breathing compartment was computed as a percentage of the sum of the three. The breathing technique of each subject was defined as pulmonary if the pulmonary compartment covered a percentage of breathing volume greater than that of the abdominal compartment. Similarly, it was defined as abdominal if the abdominal compartment covered a percentage of breathing volume greater than the pulmonary compartment. The abdominal rib cage compartment was never found to be the compartment with a highest percentage.

Secondly, a correlation analysis was performed to reduce the number of variables in the analysis. Every pair of variables presenting an absolute value of the Pearson correlation coefficient superior to 0.9 was identified as highly correlated and therefore, one of the two variables was excluded from the analysis.

#### 2.5.1 Multivariate analysis of variance

The experiment was originally designed to perform repeated measurement tests among the four combinations of the *within factors*, **state** (standard and relaxed) and **eyes** (open and closed) and compare it with the *between factor* **sex** (male and female) (Tassani et al., 2019). However, a preliminary multivariate analysis of variance (MANOVA) for repeated measures showed no effect of the *within factors* over the breathing percentages of the pulmonary rib cage, abdominal rib cage, and abdominal compartment (*p*-value >0.40) nor their interactions (*p*-value >0.29). For this reason, in the presented analysis, results of the within variables were pooled, and the capability of each subject to breathe or not abdominally was analyzed as an independent factor, **breathing**.

On the other hand, the between-factor sex was significant, with females showing a higher percentage of pulmonary volume (Estimated marginal means: female 50.24 ± 2.1, male 41.18 ± 1.9), with only 8 abdominal acquisitions over 35.

Therefore, a MANOVA was performed to analyze the relationship between 3 factors and the three sets of dependent variables: stability, posture, and emotional data. The four factors under study were the state of the eyes (**eyes**: open or closed) posture stance (**stance**: standard or relaxed) and **breathing** (abdominal or pulmonary). Sex (male, female) where analyzed separately.

#### 2.5.2 Classification

Binary logistic regression was applied to explore the possibility of classification of the breathing style of each subject starting from stability and postural data. Forward conditional feature selection was used.

#### 2.5.3 Multiple linear regression

Both MANOVA and classification analysis assumed that it was possible to divide the breathing style into abdominal and pulmonary. Multiple linear regression analysis was performed to evaluate the possible continuous relationship between breathing, postural, stability, and emotional data.

#### 2.5.4 Continuous time evaluation: statistical parametric mapping

Skin conductivity, temperature, heart rate, and acceleration of the head in three dimensions, were time-variant and therefore a time-continuous approach was proposed for their analysis. Statistical Parametric Mapping (SPM) refers to hypotheses testing of a spatially extended statistical procedure. SPM implements random field theory (Brett et al., 2003) and allows for the statistical identification of significant differences within time-variant variables.

Two-sample (independent) t-tests, performed using the MATLAB implementation “spm1d” (Pataky, 2012), were applied to examine the effects of breathing over each of the mentioned continual data.

## 3 Results

Abdominal breathing proved to be a difficult task for most subjects involved in the study. No significant difference in the use of breathing compartments was identified during the different phases of analysis (*p*-value = 0.404), showing an inability of subjects to breathe abdominally when instructed. Over a total of 81 valid breathing acquisitions, only 28 showed a dominant abdominal component (34%). Further, female subjects had greater difficulty breathing abdominally when instructed than male subjects as only 8 abdominal-dominant acquisitions (25%) were recorded from female subjects (Table 3).

**TABLE 3:** Distribution of abdominal and pulmonary breathing of male and female subjects and their percentage over the total number of acquisitions.

Sex	Abdominal		Pulmonary		Total
**Male**	20	41%	29	59%	49
**Female**	8	25%	24	75%	32
**Total**	28	35%	53	65%	81

Due to this disparity, male and female subject data were separated for subsequent analyses. Moreover, given the low number of female subjects able to perform abdominal breathing, MANOVA and classification analysis were only performed on data from males, while regression analysis was performed on both sets of data.

Twenty subjects filled out the survey collecting emotional data. Results showed the general emotional situation of subjects was critical. Seven subjects showed high anxiety (STAI>44—Ercan et al., 2015) of which, four also showed low levels of self-esteem (RSE<25—Isomaa et al., 2013). STAI and RSE were found to be negatively correlated (*R* ^2^ = 0.47, *p*-value<0.001). MANOVA and classification study were not performed with this data due to the small sample size once male and female data were separated and given the unbalanced distribution of breathing capabilities in females.

Finally, 3 MANOVA analyses were performed: for stability, divided between spatio-temporal and frequency parameters, and posture variables. To maintain the family-wise error at 5%, *p*-values were considered significant at a level of 0.017.

### 3.1 Breathing and stability

From the initial 37 stability parameters, 9 were found to be highly correlated with at least one other parameter—therefore, they were removed from the analysis. The remaining parameters in the analysis are shown in Table 1. Eighty-one acquisitions were finally valid for this analysis (46 for males and 35 for females).

MANOVA did not identify any factor or interaction related to stability for spatio-temporal or frequency parameters (Table 4). However, binary logistic regression was able to properly classify 76% of male acquisitions as belonging to abdominal or pulmonary breathing groups using only three stability variables: Standard deviation of the Transversal COP displacement, Peak number, and Peak Amplitude. Subjects showing abdominal breathing presented a reduced value of the three parameters therefore suggesting higher stability. All of them are related to the time-domain. The confusion matrix is shown in Table 5.

**TABLE 4:** *p*-values of the MANOVA analysis of Stability and Posture variables.

Factors	Stability		Posture
	Spatio-temporal	Frequency	
State	0.596	0.489	0.042
Eyes	0.342	0.293	0.506
Breathing	0.027	0.030	0.322
State * Eyes	0.528	0.640	0.978
State * Breathing	0.819	0.451	0.662
Eyes * Breathing	0.984	0.615	0.901
State * Eyes * Breathing	0.430	0.820	0.970

**TABLE 5:** Confusion matrix of breathing classification based on stability and posture variables. States of abdominal and pulmonary breathing were classified starting from, respectively, stability information based on the analysis of the COP displacement, and postural data obtained using stereophotogrammetric tools.

			Predicted stability			Predicted posture		
			Breathing		Percentage correct	Breathing		Percentage correct
			Abdominal	Pulmonary		Abdominal	Pulmonary	
**Observed**	Breathing	Abdominal	14	6	70.0	17	3	85
		Pulmonary	5	21	80.8	4	21	84
	Overall Percentage				76.1			84.4

Multiple regression analysis showed a significant relation between the use of abdominal rib cage and stability parameters in women (adjR^2^ = 0.36). For males, each chest compartment showed a relation with different stability parameters. In particular an opposite trend was identified between abdominal rib cage (adjR^2^ = 0.528) and abdominal percentages (adjR^2^ = 0.337). In males, an increased percentage of the abdomen was related to increased stability. Detailed results are shown in Table 6.

**TABLE 6:** Significant multiple linear regressions. The list of stability and postural predictors is reported along with the B coefficients and the adjusted *R* ^2^ of each of the regressions. Each set of predictors was related to the used percentage of the three chest compartments: Pulmonary, Abdominal rib cage and Abdomen. Regressions are shown separately for female and male subjects. For a complete description of the variables and their acronyms, refers to Tables 1, 2.

		Pulmonary			Abdominal rib cage			Abdomen		
		Predictors	B	Adj *R* ^2^	Predictors	B	Adj *R* ^2^	Predictors	B	Adj *R* ^2^
Female	Stability	-	-	-	(Constant)	22.968		-	-	-
					Peak amplitude (s) (m)	−1.551	0.355			
					Trasversal COP displacement(mm)(m)	0.088				
	Posture	(Constant)	60.944		(Constant)	18.924		(Constant)	14.086	
		RHPIE	−1.000	0.64	RKIE	0.509	0.865	RHPIE	1.546	0.772
		RAIE	0.701		RFAA	0.598		RAIE	−1.152	
		LKAA	1.117		SHTILT	0.160		LKAA	−2.108	
					SHROT	0.129				
	Emotion	-	-	-	-	-	-	-	-	-
Male	Stability	(Constant)	37.137	0.074	(Constant)	−37.645	0.528	(Constant)	131.157	0.337
		D spectrum, mean values Freq	18.819		Peak number	0.222		Peak number	−0.336	
					Peak amplitude (s) (m)	0.691		Peak amplitude (s) (m)	−1.011	
					Inertial axises(mm)(asseY)	0.398		Trasversal COP displacement (mm)(sd)	−0.845	
					Peak time (s) (sd)	26.986		Peak time (s) (sd)	−48.202	
	Posture	(Constant)	40.950	0.172	(Constant)	32.941	0.834	(Constant)	34.062	0.248
		RPROT	−0.438		LAFE	0.847		RHPAA	1.135	
					RAIE	−0.088				
					RKIE	0.294				
					SPFE	0.177				
					RFAA	−0.217				
					SPIE	−0.440				
					LHPAA	−0.913				
					RAFE	0.283				
					POBLI	−0.674				
	Emotion	-	-	-	(Constant)	12.157	0.506	(Constant)	56.219	0.468
					Score STAI	0.253		Score STAI	−0.466	

**FIGURE 4:**
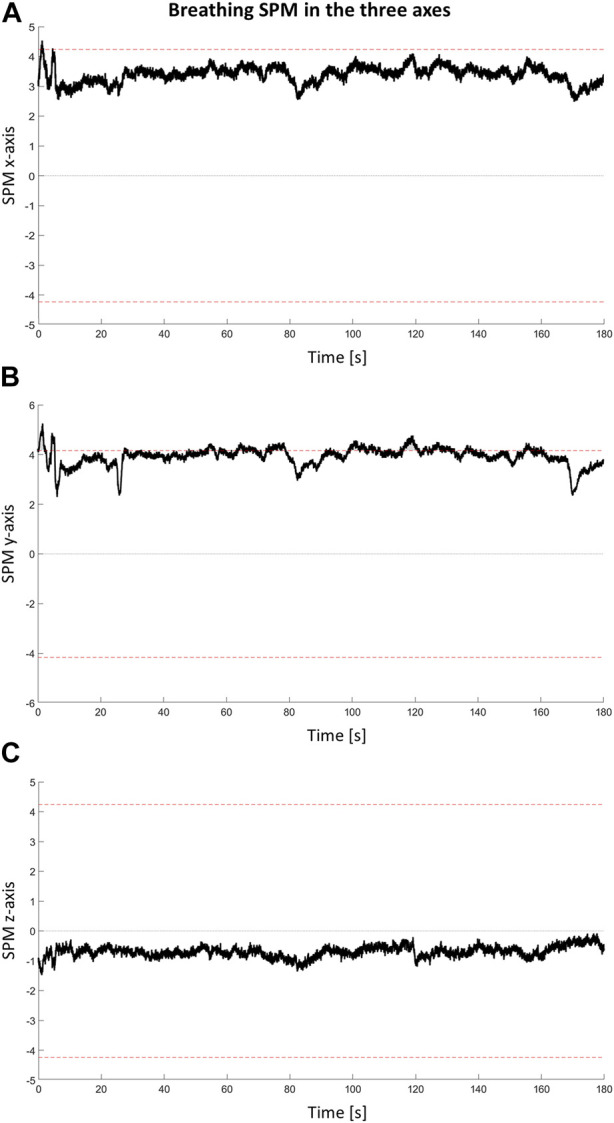
Statistical Parametric Mapping (SPM) of acceleration data. **(A)** X, **(B)** Y and **(C)** Z SPM accelerations are shown. Red dotted line identifies the significant level. When the SPM cross the red line a significant cluster is identified.

SPM of in-ear acceleration data showed statistical differences between subjects breathing abdominally or pulmonary in axes X and Y (Figure 4). Acquisitions identified as related to abdominal breathing showed reduced acceleration compared to those related to pulmonary breathing.

### 3.2 Breathing and posture

From the initial list of 29 postural parameters, 3 were found to be highly correlated with at least one other parameter—therefore, they were removed from the analysis leaving 26 parameters in the analysis (Table 2). In particular, both right and left hip flex-extension were found to be highly correlated (r > 0.9) to pelvis tilt, and spine flex-extension correlated to shoulder tilt. Seventy-three acquisitions were finally valid for this analysis (45 for males and 28 for females).

MANOVA did not identify any factor or interaction related to posture (Table 4). However, binary logistic regression was able to properly classify 84% of male acquisitions as belonging to abdominal or pulmonary breathing groups using only four postural variables: RKIE, POBLI, RHPAA, SHTILT. The confusion matrix is shown in Table 5.

Multiple regression analysis showed the relation between the use of any chest compartment and the posture of both male and female subjects. The compartment showing less relation to the posture was the pulmonary one (adjR^2^ = 0.64 for females and 0.172 for males), while the abdominal rib cage compartment showed high adj *R* ^2^ for both male (adjR^2^ = 0.834) and female (adjR^2^ = 0.865) subjects. However, the two multiple regressions differ between sexes. In female subjects, a strong relation between the use of abdominal compartment and posture was also reported (adjR^2^ = 0.772). Detailed results are shown in Table 6.

### 3.3 Breathing and emotion

**FIGURE 5:**
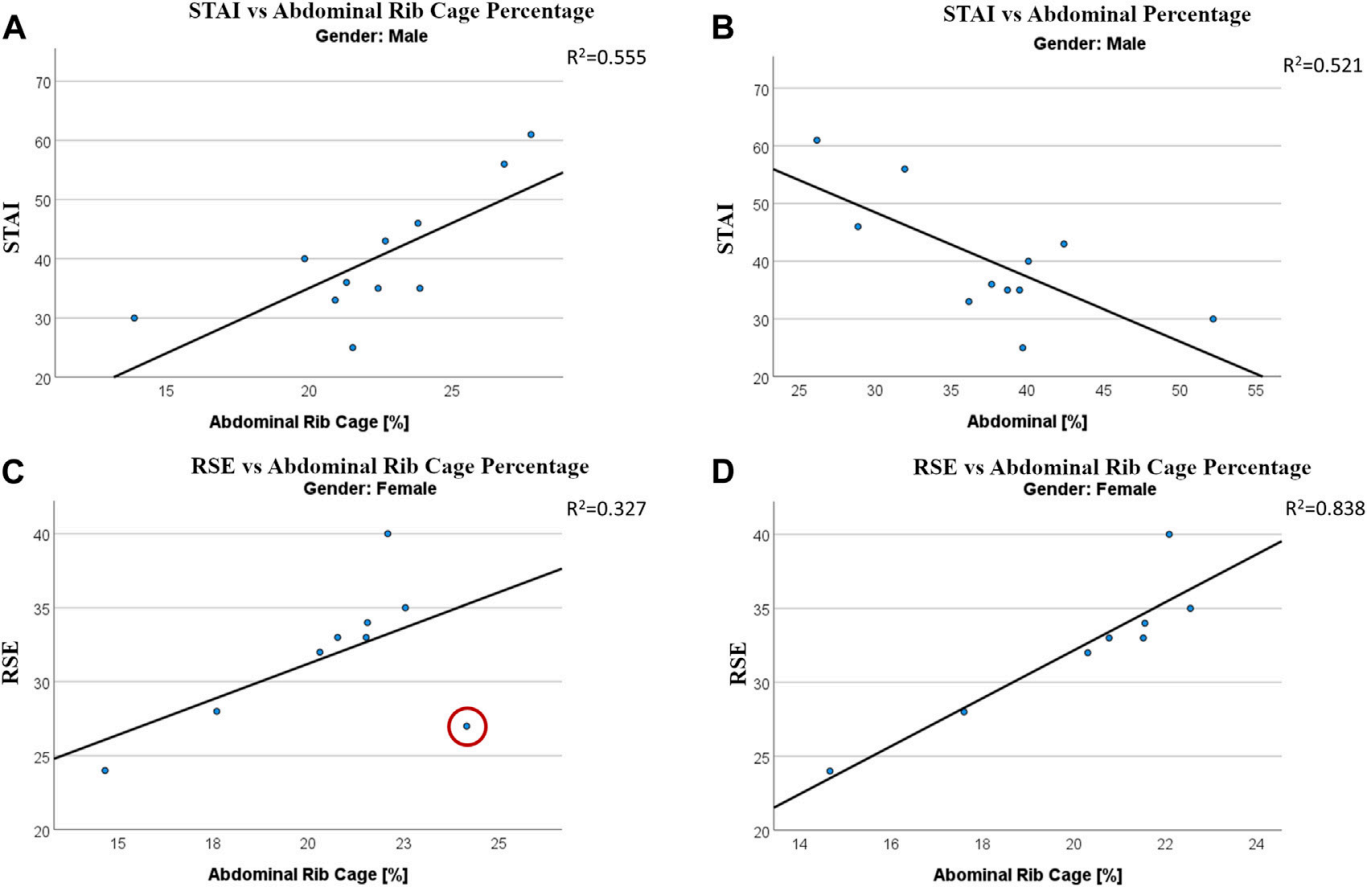
Regressions between emotional states and use of breathing compartments. Regression between State-Trait Anxiety Inventory (STAI) score and Abdominal Rib Cage **(A)** and Abdominal Compartment **(B)** in males. Regression between the Rosemberg Self-Esteem score and Abdominal Rib Cage in female, with **(C)** and without **(D)** the inclusion of a strong outlier.

The first part of the emotional evaluation was carried out using surveys, therefore it was not possible to relate these results to all four combinations of state and eyes used to acquire breathing patterns. Hence, the analysis was carried out by relating the emotional evaluation to breathing results from the condition of “standard open eyes.” This forced a reduction in sample size to 20 acquisitions, only one for each subject (11 males and 9 females). Female subjects did not report any significant multiple regression with breathing compartments while males reported significant regressions with both Abdominal rib cage and Abdominal percentage (Table 6). STAI score was found to be positively related to the use of the abdominal rib cage and negatively related to the use of the abdominal compartment (Figure 5).

SPM of both the in-ear sensor and the GSR identified no statistical difference related to the use of different breathing compartments.

### 3.4 Stability and emotion

**FIGURE 6:**
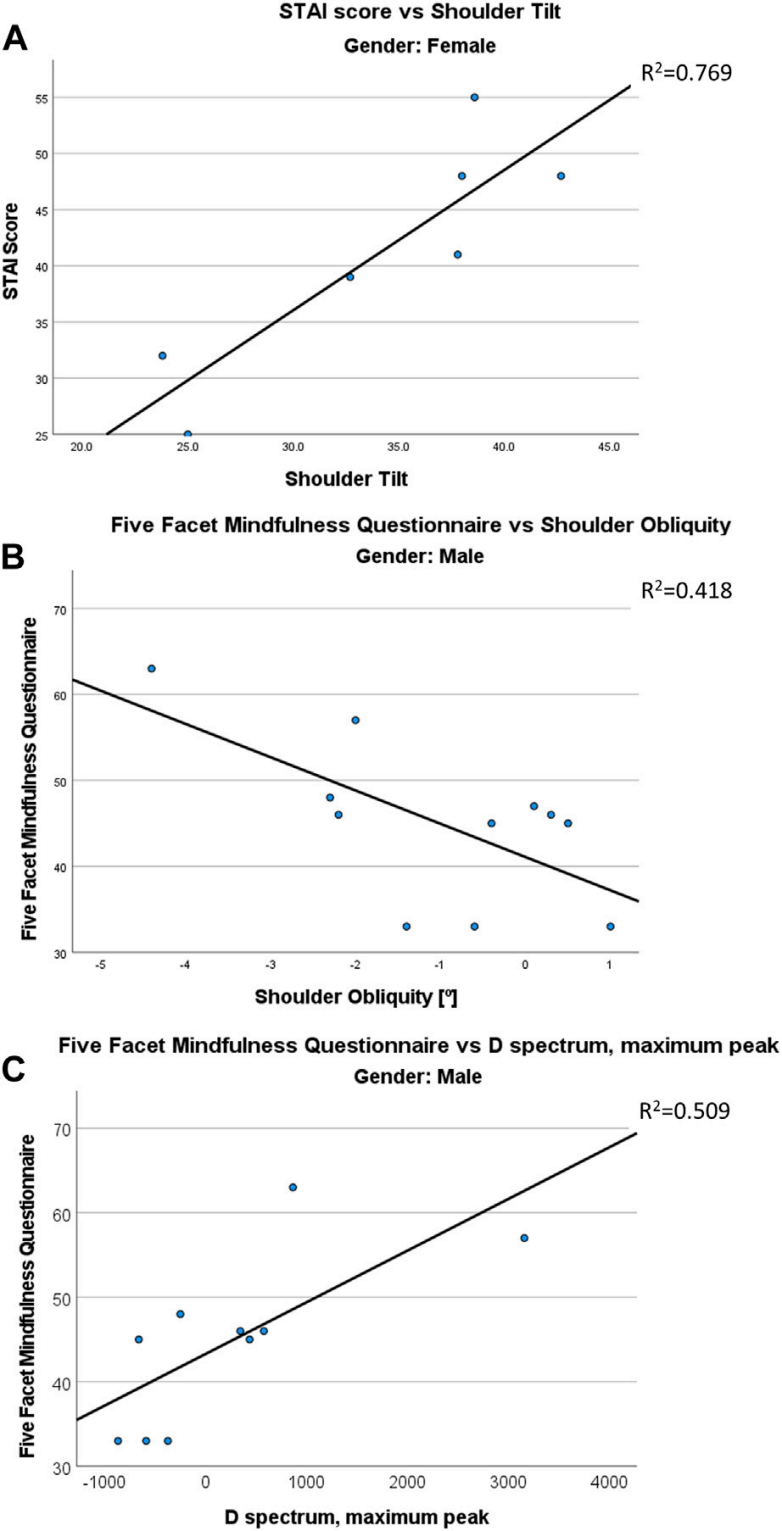
Regressions between emotional states and stability/postural parameters. Regression between State-Trait Anxiety Inventory (STAI) score with Shoulder Tilt in females **(A)**. Regression between the Five Facet score and the shoulder obliquity **(B)** and with Max peak of the spectrum of the distance from the barycenter **(C)** in males.

As for the connection to breathing, the linear relation between stability and emotional scores was studied only for the results related to the state of “standard—open eyes.” Twenty acquisitions (11 males and 9 females) were available for this analysis. Women did not show any significant relation between stability predictors and any of the emotional scores used in this study. On the other side, males presented stability relations to both STAI score (adjR^2^ = 0.727) and 5 Facet score (adjR^2^ = 0.353). Results are summarized in Table 7. The mono-dimensional regression between D spectrum and 5 Facet score in males is presented in Figure 6.

**TABLE 7:** Significant multiple linear regressions. The list of stability and postural predictors is reported along with the B coefficients and the adjusted *R* ^2^ of each of the regressions. Each set of predictors was related to the three emotional scores: the State-Trait Anxiety Inventory (STAI), Rosenberg Self-Esteem Scale (RSE), and Five Facet Mindfulness Questionnaire (5Facet). Regressions are shown separately for female and male subjects. For a complete description of the variables and their acronyms, refers to Tables 1, 2.

		STAI			RSE			5Facet		
		Predictors	B	Adj R^2^	Predictors	B	Adj R^2^	Predictors	B	Adj R^2^
Female	Stability	-	-	-	-	-	-	-	-	-
	Posture	(Constant)	−1.372	0.723	(Constant)	57.062	0.978	-	-	-
		SHTILT	1.247		SHTILT	−0.486				
					RAIE	1.242				
					RHPIE	−0.370				
Male	Stability	(Constant)	163.207	0.727	-	-	-	(Constant)	43.272	0.448
		Regression angle:()	0.188					D spectrum, maximum peak	0.006	
		Peak time (s) (sd)	−234.857							
		D spectrum, maximum peak	0.005							
	Posture	(Constant)	31.246	0.840	(Constant)	26.545	0.832	(Constant)	41.078	0.353
		RHPIE	−0.385		RKFE	1.599		SHOBLI	−3.872	
		LAIE	−1.018		LAFE	−1.484				
		RHPAA	−1.317		RKAA	−0.721				

### 3.5 Posture and emotion

As for the connection to breathing, the linear relation between posture and emotional scores was studied only for the results related to the state of “standard—open eyes.” Eighteen acquisitions (11 males and 7 females) were available for this analysis.

Women showed significant correlations between postural parameters and both STAI (adjR^2^ = 0.723) and RSE (adjR^2^ = 0.978) scores while men showed significant correlations between postural parameters and all emotional ones (Table 7).

## 4 Discussions

The proposed protocol allowed for the simultaneous analysis of breathing, postural stability, and emotional indicators. Although the high number of reflective markers used for the analysis required a lengthy preparation time (around 30 min), the protocol allowed us to test the relations between breathing, postural stability, and psychological health, observing significant results and initiating the development of a relational map among the main sets of variables in the study.

Subjects showed general difficulties in performing abdominal breathing. The majority were not able to change their breathing pattern when requested, confirming previously reported findings (Tassani et al., 2019). A few minutes of training before acquisitions were not sufficient for subjects to learn to perform abdominal breathing intentionally. This result is consistent with the literature. Studies assessing the effects of abdominal breathing on a pathology found that deep breathing training phases vary from 3 weeks to 56 months in asthma (Santino et al., 2020). It is, therefore, reasonable to consider that subjects unable to perform deep breathing could not learn to do so during a brief session of a few minutes.

**FIGURE 7:**
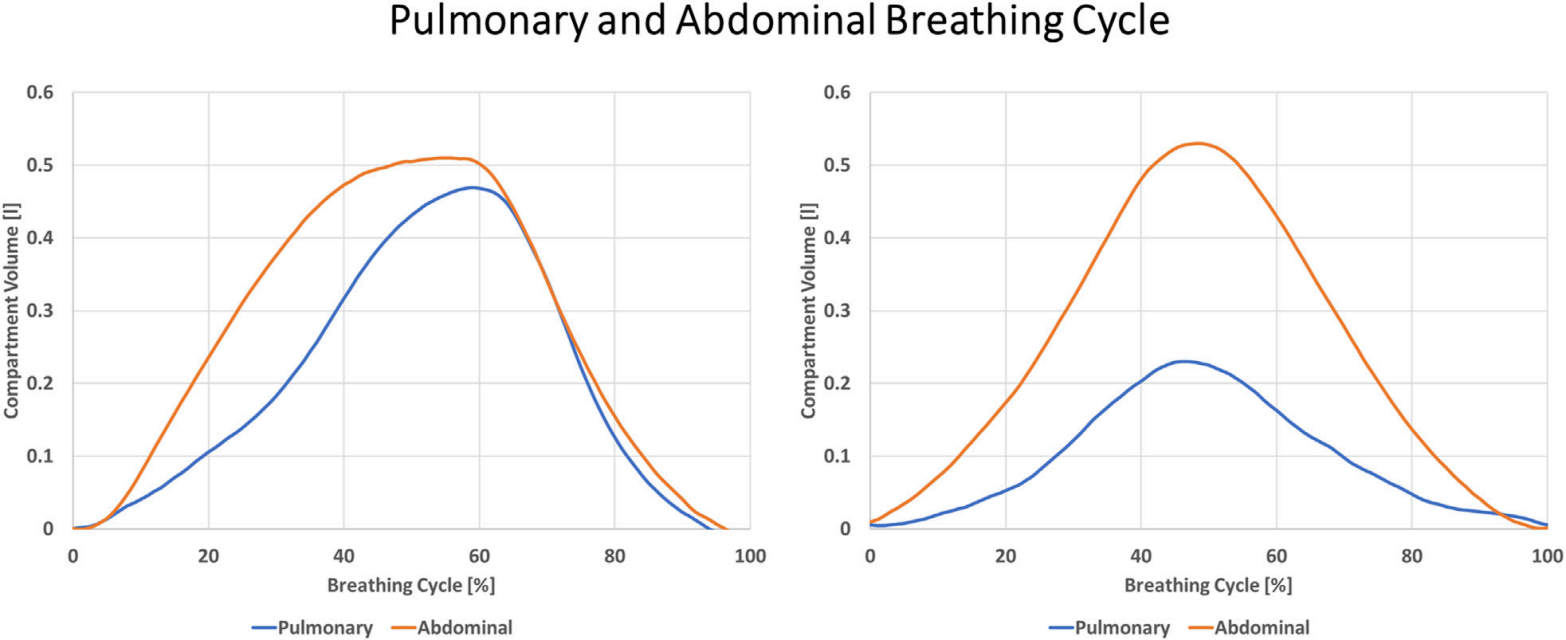
Two different breathing patterns are presented. On the left, Abdominal and Pulmonary patterns are similar with a small prevalence of abdominal breathing. On the right, the abdominal compartment is dominant over the whole breathing cycle. Both patterns were classified as Abdominal.

In this study, some subjects were constantly breathing abdominally or pulmonary and kept the same pattern for all four acquisitions. They only changed the total volume inhaled. In other cases, subjects were breathing abdominally at first but changed to pulmonary when asked to perform deep abdominal breathing. Subjects did not seem capable of controlling their breathing pattern intentionally. Only one subject was able to start deep abdominal breathing when requested. Furthermore, it must be considered that the threshold defined for the definition of abdominal breathing was arbitrary. In this study to define a breathing pattern as abdominal, the abdominal compartment must be used more than the pulmonary one. When a more conservative threshold for deep breathing classification is applied, in which the abdominal compartment is used for 60% of the abdominal and pulmonary volumes together, the number of acquisitions classified as abdominal would drop to only 5 (data in the Supplementary Material). This forced the use of a less strict threshold to classify rather different patterns as abdominal (Figure 7).

Given the importance that literature attributes to the ability to control breathing patterns for both physical (Hodges and Gandevia, 2000; Gilbert, 2003; Hodges et al., 2007) and emotional (Perciavalle et al., 2017; Hopper et al., 2019; Balban et al., 2023) management, this study presents a worrying scenario for young university students. Worries were confirmed by the number of subjects presenting high levels of anxiety in this study. The situation might be even more critical for female subjects who showed less capability of abdominal breathing (Table 3) and presented more cases of high anxiety (4 females and 3 males).

The reasons for this disparity between male and female subjects are not clear. The effect of sex on chest wall kinematics in literature is controversial. In agreement with our results, some authors have reported a relatively lower abdominal contribution to tidal volume and smaller abdominal dimensional changes in females compared to males during quiet breathing (Fugl Meyer, 1974; Gilbert et al., 1981; Romei et al., 2010). Others did not find any sex-related differences in thoraco-abdominal motion during quiet breathing in different postures (Sharp et al., 1975; Verschakelen and Demedts, 1995). However, the authors would like to stress the importance of not restricting the study of causes of this difference to biological ones. Sociological causes such as differences in gender beauty canons can lead to different perceptions of abdominal breathing and should also be explored.

Differences in behavior between sexes are another result of this study. In fact, while breathing was found to be related to both physical and emotional variables, these relations were found to be different between the two sexes. The analysis of individual indicators and their meaning was not the aim of this study; however, specific patterns can be identified and are for future research to be confirmed.

Postural characteristics were shown to be strongly related to breathing for both sexes (Table 6). The linear regression analysis showed a strong linear relation of abdominal rib cage and abdomen percentages to postural parameters. In males, postural parameters correctly classified 84% of acquisitions as abdominal or pulmonary (Table 5). This is an interesting result since posture parameters also showed the highest link to emotional scores, especially STAI and RSE, for both males and females (Table 7). These findings reinforce results obtained in the relation between breathing and emotional states and suggest the role of breathing as a mediator.

The results of the stability analysis presented weaker relations, especially in females. In males, MANOVA showed no significant results, however, the breathing factor was close to significance for both spatio-temporal (*p* = 0.027) and frequency (*p* = 0.03) parameters. In the regression analysis, both abdominal rib cage and abdomen percentages were related to stability parameters. Three out of four predictors selected by the stepwise procedure were the same, but the coefficients were inverted. This result suggests that, while an increase in the use of the abdominal rib cage compartment was related to the decrease in stability, the increased use of the abdominal compartment increased stability. SPM results of accelerometer data were found to be in the same direction. In several moments along the 3-min acquisition, subjects classified as using abdominal breathing showed a reduced acceleration of the head, suggesting higher stability (Figure 4). This supports the idea that a relaxed posture, decreasing co-contraction, can increase subject stability (Tassani et al., 2019). In males, stability was also found to be related to emotional states, whereas an increase in STAI score was related to a decrease in stability.

The main limitation of this study is in its aim of presenting a holistic protocol for the study of a breathing-physical-emotional relation. The physical sphere was analyzed in the form of stability and posture, and the emotional sphere in terms of anxiety and self-esteem, and for both measurement and analysis protocols had to be defined. For this reason, statistical analyses were presented as parts within a whole protocol. Results must be considered with caution and confirmed by future studies involving a bigger sample size. This limitation becomes clearer in the relation between breathing and emotional scores in females. In Figure 5 we can see how a single bivariate outlier can change the relation between RSE score and Abdominal Rib Cage percentage from not significant to *R* ^2^ = 0.838. Nonetheless, this study allows us to underline difficulties and criticisms that, based on this research and presented literature, might be common to many young university students.

In conclusion, this study shows how difficulties in performing deep abdominal breathing can be related to elevated anxiety scores and decreased stability, depicting a circular self-sustaining relationship that can decrease quality of life, undermine learning, produce muscular co-contraction and, in the long term, lead to the development of musculoskeletal disorders. While holistic techniques are more frequently appearing in literature to address these problems, the presented protocol can be used to quantify the effect of such techniques on subjects.

## Data Availability

The original contributions presented in the study are included in the article/Supplementary Material, further inquiries can be directed to the corresponding author.
